# Enhancing confidence in complex health technology assessments by using real-world evidence: highlighting existing strategies for effective drug evaluation

**DOI:** 10.1186/s12874-025-02683-2

**Published:** 2025-11-03

**Authors:** Alison Antoine, Katia Desroziers, Julien Dupin, David Pérol, Rémy Choquet

**Affiliations:** 1 Roche, 4 Cr de l’Île Seguin, Boulogne-Billancourt, 92100 France; 2 Centre Léon Bérard, Clinical Research, Lyon, France; 3 Janssen-Cilag, Issy-les-Moulineaux, France

**Keywords:** Real-world evidence, Randomised clinical trials, Drug evaluation, Health technology assessment, Methodology, Health policy.

## Abstract

Randomised controlled trials (RCTs) are the gold standard for evaluating new therapies but have limitations, notably in terms of external validity. Real-world data (RWD) studies could complement RCT evidence. However, a consensus has not yet been reached on situations where RWD could offer rigorous complementary evidence to an RCT when evaluating the effectiveness of therapeutic innovations. This research aims to: (1) propose a categorisation of complex clinical situations; (2) classify the real-world evidence (RWE) approaches to be used in each situation to help reduce uncertainties or provide further evidence in drug benefit assessments; (3) summarise the best methodological considerations to adopt when using these RWE approaches; and (4) propose general recommendations to increase confidence in the use of RWE approaches during the assessment process. The main recommendations within the framework around the RWD-generation plan for complex evaluations are related to four main issues: quality (establishing criteria and standards for quality data), methodology (ensuring the use of the best methodological approaches), transparency (from the industry and from the health technology agencies (HTAs) and sharing/collaborating across countries and HTAs (promoting collaboration between HTAs and involving all parties). Our proposal and recommendations could help the scientific community better consider the therapeutic value of innovations through RWD, so that their potential can be fully realised to benefit the quality of care and the regulation of the healthcare system.

## Background

While randomised controlled trials (RCTs) remain the gold standard for providing evidence when evaluating a new therapy, several limitations are associated with their use including a lack of external validity. Real-world data (RWD) studies are increasingly being used to supplement the body of evidence generated by randomised or alternative clinical trials [1]. The Food and Drug Administration (FDA) defines RWD as data relating to patient health status and/or the delivery of health care routinely collected from various sources. Real-world evidence (RWE) refers to clinical evidence regarding the use of a medical product and potential benefits or risks derived from the analysis of RWD. RWD is currently used in epidemiology for evaluating disease burden and monitoring new therapy effects in real-world (RW) conditions. This includes integrating such data into care strategies and determining their impact on healthcare organisations. However, there is less consensus on situations where RWD could robustly complement RCT evidence when evaluating the effectiveness of therapeutic innovations [2]. 

Drawing on a targeted literature review and the statistical expertise of the authors as well as their experience in submitting dossiers and managing atypical clinical development scenarios, this experience-based review aims to: (1) propose a categorisation of complex clinical situations (Table 1); (2) classify the RWE approaches to be used in each situation to help reduce uncertainties or provide further evidence in drug benefit assessments (Table 2); (3) summarise the best methodological considerations to adopt when these RWE approaches are used (Table 2); and (4) propose general recommendations to increase confidence in the use of RWE approaches during the assessment process.

**Table 1:** Complex and limiting clinical situations

	Complex Clinical Situations		
	Limited External Validity of RCTs	Treatment Comparison Issues	Non-Standard Endpoints
**Limitation(s)**	**Generalizability and transportability issues: when the treatment effect estimated in the clinical trial is not transportable to the real-world target population.** ● More restricted RCT population than local intended population ● Broader RCT population than local intended population ● Countries not represented in the international RCT	**Comparison issue: when the ideal gold standard cannot be conducted.** ● Single-arm study not allowing a controlled (counterfactual) approach ● Controlled study with non-clinically relevant comparator ● Clinical trial comparator not (or no longer) relevant at the time of evaluation (for competitive landscape for example) ● Important population heterogeneity in flexible design (multiple indications, disease severity, between patients…). E.g.: basket/umbrella/platform trials **Comparison issue: when other critical situations may affect internal validity.** ● The protocol has undergone a major amendment impacting the primary endpoint ● Crossover is permitted without intermediate analysis ● Non double-blind/open-label study	**Endpoint validity issue: uncertainty in the clinical effect and validity of the main clinical criteria.** ● Primary RCT clinical endpoint not recognised as gold standard. E.g.: surrogate endpoint, additional outcome measures such as digital or biomarkers, PREMS/PROMS indicators ● The outcome of interest is only secondary and not taken into account in hierarchical design or as a co-criterion
**Why This Situation?**	● Geographic differences in international RCTs (between-country/region), healthcare system differences, and ethnic differences ● Lack of knowledge at the time of trial design that led to a missing stratification factor in the RCT ● Heterogeneous treatment effect sizes within specific subgroups where initial design did not provide for calibrated subgroup analyses	● Unethical control arm in Phase II (e.g., gene therapy, loss of chance, very advanced last-chance cancer situations) ● Control arm not feasible in a reasonable time frame: recruitment difficulties in rare diseases/mutations ● A new drug supplanting the control arm and becoming the standard-of-care within the fast-tracking of concomitant new-drug development	● Rationale for surrogate endpoint use: long trial durations are required to demonstrate long-term efficacy on a gold standard criterion (e.g., OS in oncology) ● Rationale for additional endpoint use: medical need not yet covered and/or need to evaluate the evolution of a disease in a new way ● Potential PRO issue: no valid questionnaire available at the RCT launch

*OS* Overall survival, *PREMS* Patient-reported experience measures,* PROMS* Patient-reported outcomes measures, *PRO* Patient-reported outcome, *RCT* Randomised controlled trial

**Table 2:** RWE solution: contribution of RWD and innovative methodologies to informing evaluation and decision-making in three complex clinical situations

	Complex Clinical Situations		
	Limited External Validity of RCTs	Treatment Comparison Issues	Non Standard Endpoints
**RWD Study before HTA Submission**	● Conducting an environmental observational study to describe the locally intended population ● Conducting an RWD study for transporting RCT results targeting the population of interest ● Conducting an RWD study for extrapolating RCT results to clinical practice in another country/region	● In the protocol, provide for an a priori external comparison from RWD (cohort, population-based registry, EHR) in each relevant subgroup, in the case of basket/umbrella/platform trials	● Conduct an RWD study to describe RCT outcomes in an RWD setting and evaluate the prognostic aspect of the surrogate or novel endpoint, e.g., correlation between surrogate endpoint and gold standard ● Develop a PRO if not existing or not recognised ● Perform a literature review and meta-analyses for endpoint validation
**RWD Study after HTA Submission**	● Conducting a post-launch RWD study to describe the real-world treated population, the insertion of the new approved drug in healthcare management and to support effectiveness ● Conducting a post-launch RWD study for investigating effectiveness: direct RWD comparison (pragmatic trial, emulating a target trial)	● Conducting post-launch RWD study for investigating effectiveness: external comparison from RWD and/or direct RWD comparison (pragmatic trial or emulating a target trial)	**To conduct a post-launch RWD study with the aim of**: ● Confirming the clinical efficacy observed on the surrogate or additional endpoints in the RCT ● Evaluating the effectiveness of the molecule on the gold standard endpoint ● Assessing the correlation between the surrogate or additional endpoints and the gold standard on the molecule of interest in real life
**Key Methodological Considerations to Address RWD Limitations**	● Identification of potential confounding factors (e.g., using a DAG) ● Identification of data sources that could maximise volumetry, granularity and quality: this could be challenging, especially in some diseases, due to the availability of relevant confounding factors and the proportion of missing data ● Use of advanced adjustment techniques (e.g., propensity score matching, PS-weighting, G-computation, Doubly robust estimation) ● Need for sensitivity analyses to quantify the impact of potential sources of residual bias (due to unmeasured confounding factors, due to missing data, etc.)		**Before HTA submission**: ● Evaluate the prognostic aspect of a surrogate alone will not be sufficient to prove its validity ● The development of a new PRO is a long process with several methodological considerations ● For a new therapeutic class, surrogate validation is unfeasible in the concerning product class **After HTA submission**: ● Confirmation of treatment effect in RW could be challenging if the new treatment is best-in-class and comparison is not feasible against the previous standard of care (not possible to adjust on relevant confounding factors)

*DAG *Directed acyclic graph, *EHR *Electronic health record*, HTA *Health technology agency*, PRO *Patient-reported outcomes,* PS* Propensity score*, RCT *Randomised controlled tria*, RW *Real world*, RWD *Real-world data,* RWE *Real-world evidence

In addressing the challenges of clinical research, we discuss three key areas essential to advancing evidence-based medicine. First, the limitations of RCTs, particularly their constraints in certain clinical settings, will be examined (see Table 1 on Complex and limiting clinical situations). We will then explore the contribution of RWD, emphasizing its potential to complement and enhance traditional RCT methodologies, while also highlighting key points for vigilance (see Table 2 on RWE capabilities to address complex clinical situations). Finally, we will issue recommendations tailored to specific clinical scenarios, outlining practical strategies for integrating both RCTs and RWD to optimise decision-making and improve patient outcomes.

The topics addressed are still very recent and underutilised, though they are becoming more common. To our knowledge, no comprehensive review has been conducted that combines and articulates the limitations of clinical trials with the potential of RWE, the methods that can enhance them, and the solutions to improve the submission and evaluation of HTA dossiers.

## Limitations of RCTs in complex clinical situations

In this section, we propose a categorisation of situations where research questions are still open and the use of RWD could contribute to achieving better informed and more efficient regulatory decision-making, hereafter called complex clinical situations (as detailed in Table 1).

### External validity of an RCT

The external validity of the study may be under scrutiny due to disparities in care between the controlled settings of the RCT and routine RW conditions. These disparities may manifest in various ways, including differences in treatment dosage, the use of multiple medications, adherence to medical practices and guidelines, and variations in settings such as geographical locations and healthcare facilities. Additionally, key patient characteristics such as age, comorbidities, and treatment compliance may differ between RCTs and RW populations. The stringent inclusion/exclusion criteria often used in RCTs exclude significant segments of the general population [3]. 

Consequently, the differences observed between RCTs and RW populations can contribute to variations in treatment response, highlighting generalizability, transportability, and transferability issues and potentially widening the gap between efficacy and effectiveness. The generalizability of outcomes refers to the extent to which the results of a study sample of a target population (potentially biased) can be applied to the entire target population (including the study sample). The generalisability issue arises depending on the degree to which the findings are likely to hold true beyond the specific context in which the study was conducted. The transferability and transportability of outcomes expand generalizability by considering the applicability of study results to populations that may differ significantly from the one studied. The transportability of findings requires evaluating whether the causal relationships and observed effects in one study can be effectively transported or extended to different populations or conditions, such as different cultural or socioeconomic groups, even if the sample and the new population do not overlap perfectly or at all [4, 5]. This statistical approach focuses on quantitative research and involves adjustments to apply findings to different populations by using weighting methods, outcome regression methods, or doubly robust approaches that combine both propensity score and outcome regression [4, 5]. For example, Ramagopalan et al. (2022) [6] evaluated whether overall survival estimates from a United States population receiving first-line treatment for advanced non-small cell lung cancer were transportable to a Canadian population receiving the same therapies. However, transferability is related to a qualitative assessment and relies on conceptual and theoretical reasoning to determine whether insights found in one qualitative study can be relevant and applicable to different settings, such as assessing if the findings from a qualitative study about patient experiences in a rural healthcare setting can be relevant to an urban healthcare setting [7]. 

### Issue of treatment comparison

The issue of treatment comparison can emerge when an ideal randomised controlled trial cannot be conducted, such as in single-arm studies, when comparator arms are considered irrelevant by HTAs, or in new trial methodologies that incorporate flexible designs.

Single-arm studies can be particularly useful when recruiting participants for a control arm in a comparative study, such as an RCT, proves challenging. This is often the case in rare diseases, in heterogeneous populations, and in severe or incapacitating conditions, where ethical concerns or low accrual rates may make a placebo control or standard care arm questionable, thereby hindering the demonstration of causal treatment effects. Rees et al. (2019) reported that insufficient patient accrual was the most common reason for trial noncompletion, with nearly one-third of rare disease trials between 2010 and 2012 being discontinued for this reason [8]. 

Thus, an increasing number of single-arm trials are being submitted to authorities for final evaluation or conditional evaluation before pivotal RCTs. However, as they do not rely on counterfactual reasoning, these non-comparative studies only demonstrate the value of the benefit-risk contribution of a therapeutic innovation in treated patients. Regulatory and HTAs acknowledge that having a high-quality external control arm (ECA) derived from RWD can help reduce uncertainties in single-arm trials. Jaksa et al. (2022) [9] reported that in seven case studies with ECAs to support efficacy claims in oncology, the ECA appeared to have a significant impact on only 7 out of 34 total agency assessments. However, agencies make decisions on the basis of the entirety of the evidence and do not publicly disclose which evidence held more weight in their decision-making process, making it difficult to assess the impact of the ECA on the final decision. The most common criticisms made by agencies included selection bias and confounding, with selection bias being the most frequently mentioned limitation of ECAs across multiple case studies [9]. These challenges can be mitigated through careful and appropriate data selection and robust study design (see Section 3c, Methodological considerations to overcome the limitations of RWD section).

The comparator arms may be deemed irrelevant by HTAs. Despite the fast-tracking of new drug development and approval, clinical development remains a lengthy process during which the comparators used in pivotal RCTs may have been valid at the time the protocol was drafted and aligned with recommendations [10]. However, by the time the dossier is submitted to the HTA, these comparators are often already outdated, thus potentially challenging the benefit-risk assessment.

Developing new trial methodologies for flexible designs plays an important role in advancing medical research. The innovative molecules being developed are increasingly tailored to specific patient profiles and can sometimes be highly complex. New clinical-trial designs allow the simultaneous testing of different therapies for the same indication (umbrella trials), an innovative therapy for different indications (basket trials), or different therapies for the same indication that can enter or leave the trial continuously according to pre-established rules (platform trials) [11]. However, despite the increasingly formalised framework for these new designs, they are open to some methodological criticisms. More specifically, in basket trials, in addition to lacking a control group (in light of the philosophy of the concept), the heterogeneity of the population, both in terms of disease (e.g., multiple indications/intra- and inter- tumoral) and between patients (interpatient heterogeneity), is often questioned in terms of the efficacy of extrapolation across different subgroups (or indications) [12]. 

Issues of treatment comparison can also arise in other critical situations affecting internal validity. Situations leading to potential bias and affecting clinical trial findings include: major protocol amendments during the study impacting the primary endpoint; authorisation for widespread crossover from the control to the experimental arms post-progression; non-double-blind or open-label studies without a centralised review of the primary endpoint; or studies leading to decisions on the basis of subgroup analyses performed without statistical risk control.

### Non-standard endpoints: uncertainty in the treatment effect and validity of the main clinical criteria

Given the fast-tracking of concomitant new-drug development in a specific therapeutic area, the choice of primary efficacy endpoint used in clinical trials is a major challenge, and non-standard endpoints are often prioritised over hard outcomes.

Surrogate endpoints are often used as the primary endpoint. Follow-up times in RCTs are generally limited for observing long-term survival. In oncology, in light of the pursuit of innovations and the accelerating drug development, although overall survival is one of the gold-standard endpoints in RCTs [13], only a quarter of pivotal studies submitted for European Medicines Agency (EMA) approval are calibrated to evaluate it as a primary endpoint [14]. Thus, in oncology, the survival endpoints used to obtain a marketing authorisation are often surrogate endpoints such as disease-free survival in adjuvant settings or progression-free survival in metastatic settings. However, their surrogate value for overall survival is limited [15–17] and does not provide a high level of certainty when assessing the clinical benefit of new therapeutic classes such as immunotherapies.

The emergence of novel outcome measures (e.g., digital, biomarkers, etc.) meets the demand for identifying efficacy criteria better suited to specific indications or that are more patient-centered [18–20]. However, the validation of these additional endpoints is essential before they are used to effectively demonstrate therapeutic efficacy.

PREMS/PROMS (Patient-Reported Experience Measures/Patient-Reported Outcome Measures) indicators are increasingly being considered by healthcare providers and authorities [21–23]. Nevertheless, these indicators are most often evaluated as secondary endpoints in clinical trials, without any prior research assumptions, and the statistical power of their analysis does not provide a sufficient level of evidence regarding the benefits of the intervention on quality of life or patient experience. These data can benefit ratings, but discrepancies in interpretations pose challenges in meeting the diverse requirements of all HTAs [24].

## The contribution of RWD and innovative methodologies to inform evaluation

The complementarity of RWD and RCTs can be a major driver for improving knowledge of clinical practice, decision-making, and the quality and relevance of patient care.

### RWD studies

Many RWD sources are available for many conditions, including registries, cohorts, health insurance databases, electronic health records (EHRs) and observational studies. Moreover, different sources that relate to the same population, such as an EHR-based cohort and health insurance claims data, can be linked to create a new enhanced dataset to study drug safety and effectiveness with improved data completeness and follow-up. These data are collected from larger and more heterogeneous populations than RCTs and enable prolonged follow-up, which is particularly suited to the evaluation of overall survival.

Environmental observational studies can add to our knowledge of the epidemiology, natural history of the disease, patient care, and even disease burden, irrespective of regulatory time frames. RWD studies can also be used to identify and describe the target population of an indication, identify prognostic risk factors for disease development, identify and validate clinically relevant endpoints (e.g., surrogate endpoints), or identify subpopulations of interest. As for comparative observational studies, RWD studies can be designed to compare a treatment in routine practice to a clinically relevant comparator, which is not considered obsolete or uncompetitive. The effectiveness in populations exposed to the treatment in routine practice can also be evaluated against several therapeutic strategies under RW conditions and not only against the active comparator, as in an RCT.

### Limitations of RWD studies

While observational data may offer high external validity owing to their representativeness of RW conditions, the presence of various biases hampers their internal validity and can limit their ability to establish causal relationships [25].

Notably, selection bias [26] arises from the observational nature of RWD since the disease risk factors are not randomly assigned to the groups of subjects being compared. Consequently, in the absence of randomisation, it is impossible to guarantee the comparability of patients included in each of the groups. The unequal distribution of treatment chances (indication bias) is also logical since individuals are not assigned randomly to the treated or untreated group.

Furthermore, confounding bias can also occur because an association may exist not only between the choice of treatment and other factors but also between these factors and the endpoint, making it difficult to determine if the observed results are attributable solely to the intervention. Such associations must be taken into account to minimise the risk of confounding as much as possible [27].

When comparing two arms, the lack of randomisation can also result in a misalignment of the start times (Time 0) of patient follow-up, which can lead to an immortal time bias [28, 29]. Finally, the problem of missing data can also lead to bias if the available observations do not have the same properties as those of the unobserved data.

The inherent limitations of generating RWD and their use in establishing clinical evidence raise questions about methodological choices and the use of complex statistical techniques to address certain biases and improve causal inference when comparing treatment effects.

### Methodological considerations to overcome the limitations of RWD 

Innovative methodologies such as target trial emulation (TTE) have been developed to address bias in observational studies. While some HTAs and key stakeholders already recommend TTE as a best practice for designing RWE studies [30–33], the EMA [34] and the FDA [35] have recently released a reflection paper and draft guidance, respectively, that cites TTE as a strategy to increase transparency and reduce confounding and selection bias[34, 35] TTE is a recently designed methodological framework developed by Hernán et al. (2016, 2022) [36, 37] to address selection bias by applying inclusion and exclusion criteria and confounding by adjustment [36–41]. The aim is to apply a target RCT protocol to answer the question of the effectiveness of treatments being compared. The protocol of the target trial defines upstream seven elements: (1) eligibility criteria; (2) therapeutic strategies; (3) assignment procedure; (4) follow-up period; (5) endpoint; (6) causal effect of interest (intention to treat/per-protocol); and (7) analysis plan. To control confounding, this approach requires statistical analysis to replicate the effect of randomisation in terms of measured confounders. These analyses may use advanced confounding-adjustment techniques to balance the treatment arms such as propensity score-based methods [42], or more recent counterfactual approaches, such as G-computation (where G stands for “general”) or doubly robust estimation [43, 44]. Directed acyclic graphs (DAGs) [45] can help identify confounders relevant to the treatments being compared and avoid adjustment for factors called “colliders” or “mediators”. A TTE approach may be relevant (1) in post-registration situations to compare two treatment arms in the real world (2), and in pre-registration situations to provide an adequate comparison against an external control arm when it is impossible to conduct an RCT [4].

Specific statistical methods exist to quantify the impact of potential sources of residual bias, including unmeasured confounders and missing data. Various statistical methods provide an unbiased estimate of the treatment effect on the basis of the assumption that what is not observed will not influence the choice of treatment [47, 48]. In the absence of randomisation, since it is impossible to adjust for unmeasured and unmeasurable confounders, the issue of residual (unmeasured) bias persists.

This concern can be addressed through quantitative sensitivity analyses, such as Quantitative Bias Analyses (QBA) for unmeasured confounding [49–52]. These analyses are used to account for the impact of an unmeasured confounder on the observed treatment–outcome association (from the main analysis). They estimate how the results could be influenced by the presence of unmeasured confounding factors by estimating the relative risk that would have been obtained if an uncontrolled factor had been available for adjustment. There are various QBAs, but those applicable to the study of a survival criterion are more limited; the two most widely used approaches for exploring bias due to residual confounding are QBAs based on tipping point analysis: (i) simulation of a hypothetical unmeasured confounder and (ii) the E–value. For example, the array approach [49] tests by simulating the impact of the presence of unmeasured confounding factors under different assumptions of varying strengths of confounder–outcome associations and varying prevalence of the unmeasured confounder in the two treatment arms. The E–value approach is, however, a simplification of the tipping point analysis developed by VanderWeele and Ding (2017) [50], where the E–value is a metric representing the minimum strength of association that an unmeasured confounder would require for both the treatment and outcome to make the observed association null (i.e., relative risk equal to 1). Other more complex analyses may use negative or positive controls to help detect bias due to residual confounding [53, 54].

On the other hand, missing data are a common problem in RW studies. To prevent uncertainties about missing data for the outcome, the preferred option is to use a robust endpoint such as death. This endpoint is generally completed and is less prone to misclassification or measurement bias, and, with sufficient follow-up, can be used to measure RW overall survival. However, when some data are missing for some units of a measured variable (e.g., a measured confounder), multiple imputation, operating under the *Missing at random* assumption, addresses missing data by predicting several plausible values for each missing unit using data available for the variable and other variables [55, 56]. This repeated imputation can reduce the variability due to the prediction process. The issue of residual bias due to missing data can be further explored through sensitivity analyses and quantitative-bias analysis of missing data [57–60].

Table 2 summarises how RWD studies and innovative methodologies can help inform evaluation and decision-making in the three complex clinical situations identified.

## Recommendations

We present a set of general recommendations aimed at enhancing confidence in the use of RWE during the assessment process.

Improving the acceptance of RWD studies in the assessment of the benefit–risk balance involves several challenges, including improving RWD quality and the use of robust methodology; increasing the transparency of RWD analyses and results; the buy-in and upskilling of all parties; and a tailored and more flexible technical/regulatory assessment framework to allow the use of such data without burdening the healthcare system [61–64].

**Fig. 1:**
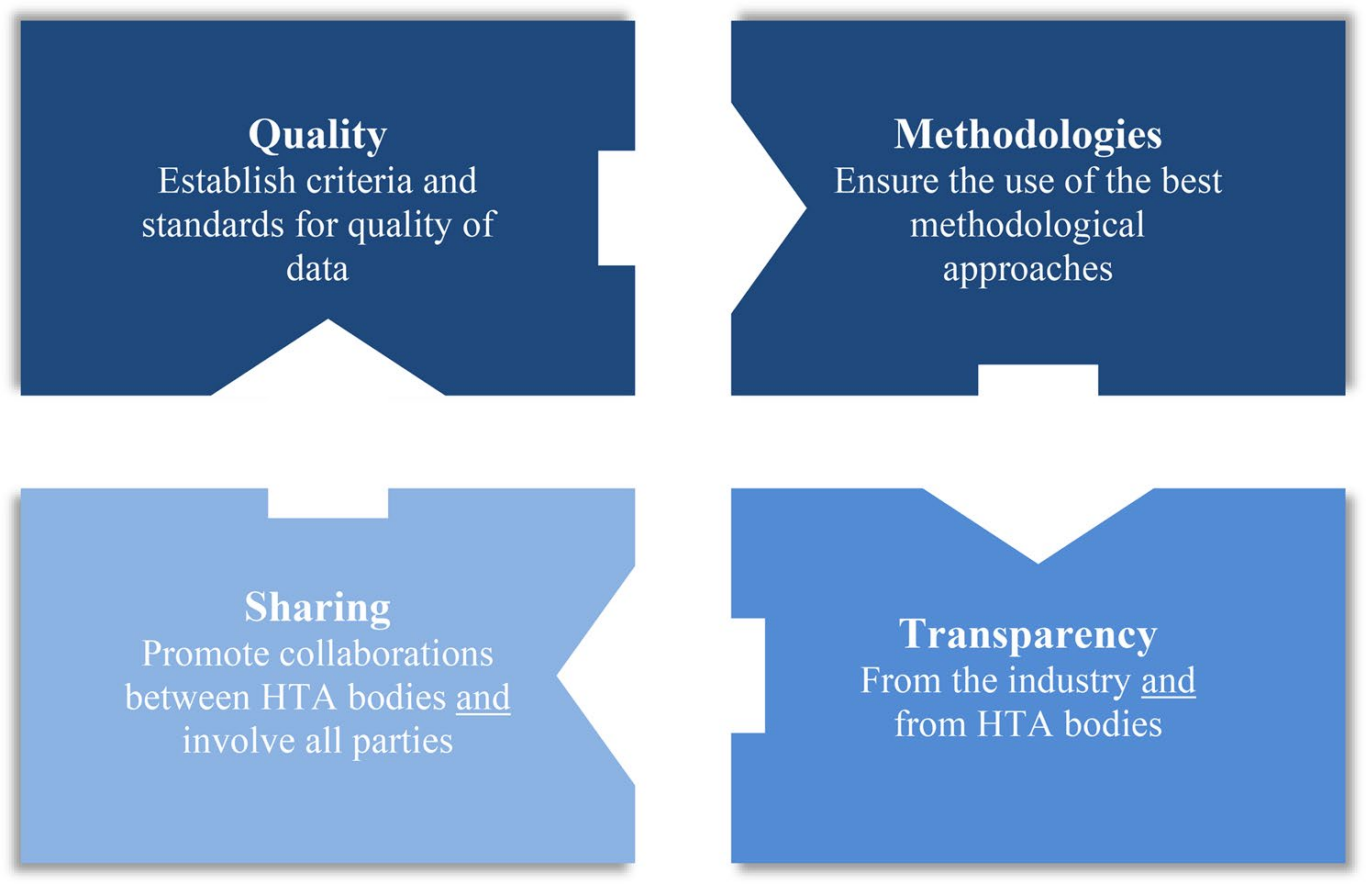
Four key areas of a framework around an RWD-generation plan for complex evaluations

In the context of using RWD for HTA decisions, solutions are available to mitigate methodological weaknesses. Solutions also exist to allow RWD integration within clinical trial developments (e.g., external controlled trials [46]) to provide further evidence of effectiveness. The following recommendations, which are based on the authors’ experience in dossier submission, could contribute to the implementation of these RWD solutions and support drug assessment and HTA decision-making processes. These recommendations are structured around four main pillars – quality, methodologies, transparency, sharing – as summarised in Figure 1 and Table 3.

**Table 3:** Main recommendations within the framework around the RWD-generation plan for complex evaluations

Four key areas
**Quality**
Establish quality qualifications and standards for RWD used in complex evaluations.
Develop a comprehensive RWD-generation plan and assessing the quality and relevance of the RWD-generation plan included in the dossier, regardless of the drug appraisal.
**Methodology**
Implement methodologies integrating the analysis of the replication of evidence across time and space.
Develop support for hearings/consultations of methodological and clinical expert panels.
Share data, programs and analysis reports.
Dedicate an assessment unit in charge of establishing quality qualifications and standards and ensure the implementation of the best methodological approach.
**Transparency**
Ensure full transparency and justification from the industry on the non-standard clinical development program.
Ensure full transparency regarding the consideration or non-consideration for the drug appraisal by the HTAs of the RWD evidence, following assessment.
**Sharing and collaborating**
Promote international exchanges between HTAs for the assessment and appraisal of these specific situations.
Promote education, teaching and research for RWD solutions and complex evaluations.

*HTA *Health technology agency, *RWD* Real world data

### Establishing quality qualifications and standards for RWD used in complex evaluations and allowing easy access

Assessing the quality of the RW database is a prerequisite for its use in HTA decision-making processes, as it is for clinical trials. Poor data quality, whether in terms of completeness, accuracy, or reliability, can introduce or exacerbate biases, particularly regarding eligibility criteria for selecting the target population and outcomes. This can unevenly affect treatment groups, leading to flawed decision-making. Criteria applicable at an international level should be tailored to the epidemiological context, and must consider volumetry, quality and granularity would address this need. It is also advisable to record the data in an official registry to allow their use for complex assessments. These steps should encourage institutional partners to provide the most fit-for-purpose data as much as possible.

Access to high-quality RWD should also be facilitated and accelerated, to ensure their use in the decision-making process, without undely burdening the health system. In this context, several initiatives, including the Data Analysis and Real-World Interrogation Network (DARWIN EU), are already in progress to increase the capacity to generate and deliver RWE across Europe [65]. Trusting third parties (such as the Health Data Hub in France [66]) should also be identified to facilitate the pooling of multiple datasets to be used for health assessments.

The establishment of quality qualifications and standards for RWD used in complex evaluations could be feasibly implemented in the short term. European initiatives in this direction, such as the NICE dataSAT or the EUnetHTA REQueST tool, have already been launched and will help standardise practices and ensure the availability of high-quality data.

### Assessing the quality and relevance of the RWD-generation plan included in the dossier, regardless of the drug appraisal

In complex situations, developing a comprehensive local or multi-country RWD-generation plan for both pre- and post-registration of medicines should be a priority for the company, ensuring it is aligned with and complements clinical development plans. This plan should be created in close collaboration with dedicated learned societies and validated through early discussions with the HTA, incorporating multipartite consultations when necessary. This would make it possible to reduce HTA uncertainties at the time of evaluation and in cases where certain uncertainties cannot be assessed, the company must ensure a robust post-HTA generation plan to resolve these uncertainties in the long term.

The various stakeholders – data providers and companies – would need to commit alongside the authorities to a realistic timetable to provide RWD linked to clinical trials or to support the clinical trial data package, prepared using appropriate methodological and clinical expertise (as detailed below). Furthermore, the results would need to be shared and discussed through regular multipartite consultations.

By employing methodologies that integrate the analysis of evidence replication across time and space, the aim is to generate a substantial number of consistent signals and ultimately provide the most reliable evidence. Evaluation of a single RW study seems unrealistic given the inherent limitations of RWD. Therefore, meta-analysis methodologies allow the integration of various types of evidence, including simulated efficacy and effectiveness data, thus combining the evidence generated by the different available studies. Consequently, HTAs could integrate into the decision-making process meta-analyses on the basis of local and international plans, providing data across time (observation periods) and space (multiple sites).

Support for hearings and consultations of methodological and clinical expert panels is key to implementing the proposed common requirements among all parties involved (e.g., manufacturers, academics, healthcare professionals, contract research organisations, and patients), including the reference methodology (cohort, register) and the type of data to be used and collected according to the diseases and indications (including local and international data). This should ensure that RWD studies are conducted properly and are able to answer issues and/or uncertainties raised by the authorities.

Sharing all the data, detailed analyses, and study reports produced by the data holders to the dedicated RWD teams of the authorities also appears to be an essential element in removing acceptability barriers, as well as enabling these teams to gain proficiency in the appropriate RWD methodologies.

A dedicated assessment unit. The integration of RWDs in an RCT (for the purpose of external comparison methods or pragmatic tests) or their use to further supplement trial data (e.g., within the emulation of RCTs) are based on innovative and complex methods that require fully fledged expertise. We recommend setting up dedicated assessment units for these complex methodologies and ensuring the relevance of the RWD-generation plan in trickier development situations. These expert units could be implemented within health authorities – such as the Committee for Economic Evaluation and Public Health (CEESP) at the French health authority (Haute Autorité de Santé, HAS), which evaluates the assessment of the cost-effectiveness model and does not interpret the level of efficiency, or as an independent group, such as the Evidence Review Group (ERG) of the National Institute for Health and Care Excellence (NICE).

Because various stakeholders are involved, assessing the quality and relevance of the RWD-generation plan included in the dossier requires a progressive implementation. 

### Full transparency and justification from the industry on the non-standard clinical development programs

The manufacturer must provide full transparency and a robust justification for any non-standard RCT design, which should be thoroughly discussed during European joint scientific consultations or early national dialogues. Support for hearings/consultations of methodological and clinical expert panels is key to confirming the rationale for “major deviations from evidence-based medicine principles”. [31]

Given the ongoing clarification of non-standard clinical development programs, [30, 31] full transparency and justification from industry are already achievable[67]and have the potential for further improvement.

### Full transparency regarding the consideration or non-consideration of the RWD evidence for drug appraisal by the HTAs, following the assessments

To ensure full transparency, HTAs should systematically provide information and rationales on the use (or non-use) of RWD studies, along with their rationale and associated impact, for the purpose of clarifying the role of RW evidence as an appraisal criterion or sub-criterion. With the aim of continuous improvement of the dossier, achieving a high level of transparency in the short term is essential.

### International exchanges between HTAs for the assessment and appraisal of these specific situations

Multi-HTA exchanges should be strengthened for these specific assessments, with the sharing or pooling of the plan to generate additional data to enable authorities to evaluate the benefit-risk balance iteratively and continuously integrate the notion of risk sharing in the appraisal and decision-making process. The reform of HTA scientific evaluation in Europe, including the two new evaluation stages at the European level, with a joint European scientific consultation and clinical evaluation, should ultimately help in this regard [68].

### Education, teaching, and research for RWD solutions for complex evaluations

All potential exchange and training channels should be explored to improve the understanding of these new methods by all parties in the ecosystem. In particular, support and training should be offered to future prescribers for the upcoming launch of health products evaluated through these RWD solutions.

The emergence of novel methods of assessing and controlling bias in RWD studies is expanding and research must continue towards the acculturation and improvement of best practices in RWD studies, especially in terms of comparative effectiveness supporting healthcare decision-making.

## Conclusions

Randomised controlled trials have limitations in a number of well-identified clinical situations. Similarly, RWD offer a valuable resource to overcome some of these limitations of RCTs. Today, the development of innovative methodological approaches and specific statistical processing allows the rigorous construction of evidence of effectiveness on the basis of high-quality RWD to supplement clinical trial data. We reviewed these novel methodological approaches and suggested ways to implement them within HTAs and decision-making processes (see Table 1 and Figure 1).

Our proposal and recommendations could also help the scientific community better consider the therapeutic value of innovations through RWD, so that their potential can be fully realised to benefit the quality of care and the regulation of the healthcare system.

## Data Availability

No datasets were generated or analysed during the current study.

## Abbreviations

CEESP: Committee for economic evaluation and public health
DAG: Directed acyclic graph
EMA: European medical agency
EHR: Electronic health record
ERG: Evidence review group
HTA: Health technology agency
NICE: National institute for health and care excellence
OS: Overall survival
PREMS: Patient-reported experience measures
PRO: Patient-reported outcomes
PROMS: Patient-reported outcome measures
PS: Propensity score
QBA: Quantitative bias analyses
RCT: Randomised controlled trials
RW: Real-world
RWD: Real-world data
RWE: Real-world evidence

## References

[1] Garrison LP, Neumann PJ, Erickson P, Marshall D, Mullins CD. Using real-world data for coverage and payment decisions: the ISPOR real-world data task force report. Value Health: J Int Soc Pharmacoeconomics Outcomes Res. 2007;10(5):326–35. 10.1111/j.1524-4733.2007.00186.x 17888097

[2] Gerstein HC, McMurray J, Holman RR. Real-world studies no substitute for RCTs in establishing efficacy. Lancet. 2019;19(10168):210–1. 10.1016/s0140-6736(18)32840-x. 10.1016/S0140-6736(18)32840-X 30663582

[3] Kennedy-Martin T, Curtis S, Faries D, Robinson S, Johnston J. A literature review on the representativeness of randomized controlled trial samples and implications for the external validity of trial results. Trials. 2015;16:495. 26530985 10.1186/s13063-015-1023-4 PMC4632358

[4] Mumford SL, Schisterman EF. New methods for generalizability and transportability: the new norm. Eur J Epidemiol. 2019;34(8):723–4. 10.1007/s10654-019-00532-3. 31175532 10.1007/s10654-019-00532-3 PMC7994943

[5] Lesko CR, Buchanan AL, Westreich D, Edwards JK, Hudgens MG, Cole SR. Generalizing study results: a potential outcomes perspective. Epidemiology. 2017;28(4):553–61. 10.1097/ede.0000000000000664. 28346267 10.1097/EDE.0000000000000664 PMC5466356

[6] Ramagopalan SV, Popat S, Gupta A, et al. Transportability of overall survival estimates from US to Canadian patients with advanced non-small cell lung cancer with implications for regulatory and health technology assessment. JAMA Netw Open. 2022;1(11):e2239874. 10.1001/jamanetworkopen.2022.39874. 10.1001/jamanetworkopen.2022.39874 PMC9634498 36326765

[7] Jaksa A, Arena PJ, Chan KKW, Ben-Joseph RH, Jónsson P, Campbell UB. Transferability of real-world data across borders for regulatory and health technology assessment decision-making. Front Med Lausanne. 2022;9:1073678. 10.3389/fmed.2022.1073678. 36465931 10.3389/fmed.2022.1073678 PMC9709526

[8] Rees CA, Pica N, Monuteaux MC, Bourgeois FT. Noncompletion and nonpublication of trials studying rare diseases: a cross-sectional analysis. PLoS Med. 2019;16(11):e1002966. 10.1371/journal.pmed.1002966. 31751330 10.1371/journal.pmed.1002966 PMC6871779

[9] Jaksa A, Louder A, Maksymiuk C, et al. A comparison of seven oncology external control arm case studies: critiques from regulatory and health technology assessment agencies. Value Health Dec. 2022;25(12):1967–76. 10.1016/j.jval.2022.05.016. 10.1016/j.jval.2022.05.016 35760714

[10] Freedland KE, King AC, Ambrosius WT, et al. The selection of comparators for randomized controlled trials of health-related behavioral interventions: recommendations of an NIH expert panel. J Clin Epidemiol. 2019;110:74–81. 10.1016/j.jclinepi.2019.02.011. 30826377 10.1016/j.jclinepi.2019.02.011 PMC6543841

[11] Park JJH, Siden E, Zoratti MJ, et al. Systematic review of basket trials, umbrella trials, and platform trials: a landscape analysis of master protocols. Trials. 2019;(1):572. 10.1186/s13063-019-3664-1. 31533793 10.1186/s13063-019-3664-1 PMC6751792

[12] Hobbs BP, Pestana RC, Zabor EC, Kaizer AM, Hong DS. Basket trials: review of current practice and innovations for future trials. Journal Clin Oncology: Official J Am Soc Clin Oncology Oct. 2022;20(30):3520–8. 10.1200/jco.21.02285. 10.1200/JCO.21.02285 PMC10476732 35537102

[13] Food and drug administration (FDA). Clinical Trial Endpoints for the Approval of Cancer Drugs and Biologics-Guidance for Industry. https://www.fda.gov/regulatory-information/search-fda-guidance-documents/clinical-trial-endpoints-approval-cancer-drugs-and-biologics

[14] Naci H, Davis C, Savović J, et al. Design characteristics, risk of bias, and reporting of randomised controlled trials supporting approvals of cancer drugs by European medicines Agency, 2014-16: cross sectional analysis. BMJ Sep. 2019;18:366:l5221. 10.1136/bmj.l5221. 10.1136/bmj.l5221 PMC6749182 31533922

[15] Prasad V, Kim C, Burotto M, Vandross A. The strength of association between surrogate end points and survival in oncology: a systematic review of trial-level meta-analyses. JAMA Intern Med. 2015;175(8):1389–98. 10.1001/jamainternmed.2015.2829. 26098871 10.1001/jamainternmed.2015.2829

[16] Haslam A, Hey SP, Gill J, Prasad V. A systematic review of trial-level meta-analyses measuring the strength of association between surrogate end-points and overall survival in oncology. Eur J Cancer. 2019;106:196–211. 10.1016/j.ejca.2018.11.012. 30528804 10.1016/j.ejca.2018.11.012

[17] Hwang TJ, Gyawali B. Association between progression-free survival and patients’ quality of life in cancer clinical trials. International J Cancer J Int Du Cancer Apr. 2019;1(7):1746–51. 10.1002/ijc.31957. 10.1002/ijc.31957 30374970

[18] Kruizinga MD, Stuurman FE, Exadaktylos V, et al. Development of novel, value-based, digital endpoints for clinical trials: a structured approach toward fit-for-purpose validation. Pharmacol Rev. 2020;72(4):899. 10.1124/pr.120.000028. 32958524 10.1124/pr.120.000028

[19] Lux MP, Ciani O, Dunlop WCN, Ferris A, Friedlander M. The impasse on overall survival in oncology reimbursement decision-making: how can we resolve this? Cancer Manag Res. 2021;13:8457–71. 10.2147/cmar.S328058. 34795526 10.2147/CMAR.S328058 PMC8592394

[20] IQVIA Institute. Evolving oncology endpoints, a new horizon for oncology outcomes. 2021.

[21] Bryan S, Davis J, Broesch J, et al. Choosing your partner for the PROM: a review of evidence on patient-reported outcome measures for use in primary and community care. Healthcare Policy | Politiques de Santé. 2014;10(2):38–51. 25617514 PMC4748356

[22] Food and Drug Administration (FDA). Guidance for Industry. Patient-Reported Outcome Measures: Use in Medical Product Development to Support Labeling Claims. 2009. 10.1186/1477-7525-4-79 PMC1629006 17034633

[23] Reflection paper on the regulatory guidance for the use of healthrelated quality of life (HRQL) measures in the evaluation of medicinal products. (2005).

[24] Chassany O, Engen AV, Lai L, et al. A call to action to harmonize patient-reported outcomes evidence requirements across key European HTA bodies in oncology. Future Oncol. 2022;18(29):3323–34. 10.2217/fon-2022-0374. 36053168 10.2217/fon-2022-0374

[25] Sherman RE, Anderson SA, Dal Pan GJ, et al. Real-world evidence - what is it and what can it tell us? N Engl J Med. 2016;8(23):2293–7. 10.1056/NEJMsb1609216. 10.1056/NEJMsb1609216 27959688

[26] Catalogue of Bias, Collaboration ND, Bankhead C, Aronson JK. Selection bias. Catalogue Of Bias 2017. Accessed 20 February 2024. http://www.catalogofbias.org/biases/selection-bias/

[27] Catalogue of bias collaboration AJ, Bankhead C, Mahtani KR, Nunan D. Confounding by indication. Catalogue Biases. 2018. https://catalogofbias.org/biases/confounding-by-indication

[28] Suissa S. Immortal time bias in pharmaco-epidemiology. American J Epidemiology Feb. 2008;15(4):492–9. 10.1093/aje/kwm324. 10.1093/aje/kwm324 18056625

[29] Lévesque LE, Hanley JA, Kezouh A, Suissa S. Problem of immortal time bias in cohort studies: example using statins for preventing progression of diabetes. BMJ. 2010;340:b5087. 10.1136/bmj.b5087. 20228141 10.1136/bmj.b5087

[30] National institute for health and care excellence (NICE). NICE real-world evidence framework (ECD9). https://www.nice.org.uk/corporate/ecd9/chapter/overview 10.57264/cer-2023-0135 PMC10690376 37855246

[31] Vanier A, Fernandez J, Kelley S, et al. Rapid access to innovative medicinal products while ensuring relevant health technology assessment. Position of the French National authority for health. BMJ Evid Based Med Jan. 2024;19(1):1–5. 10.1136/bmjebm-2022-112091. 10.1136/bmjebm-2022-112091 PMC10850619 36788020

[32] Guide on Methodological Standards in Pharmacoepidemiology; EMA/95098/2010 (Revision 11, 13 July 2023). (2023).

[33] Castelo-Branco L, Pellat A, Martins-Branco D, et al. Esmo guidance for reporting oncology real-World evidence (GROW). Ann Oncol. 2023;34(12):1097–112. 10.1016/j.annonc.2023.10.001. 37848160 10.1016/j.annonc.2023.10.001

[34] Reflection paper on use of real-world data in non-interventional studies to generate real-world evidence - Scientific guideline. (2024).

[35] Real-World Evidence: Considerations Regarding Non-Interventional Studies for Drug and Biological Products. (2024).

[36] Hernán MA, Robins JM. Using big data to emulate a target trial when a randomized trial is not available. American J Epidemiology Apr. 2016;15(8):758–64. 10.1093/aje/kwv254. 10.1093/aje/kwv254 PMC4832051 26994063

[37] Hernán MA, Wang W, Leaf DE. Target trial emulation: A framework for causal inference from observational data. JAMA: J Am Med Association Dec. 2022;27(24):2446–7. 10.1001/jama.2022.21383. 10.1001/jama.2022.21383 36508210

[38] Fu EL. Target trial emulation to improve causal inference from observational data: What, Why, and how? Journal Am Soc Nephrology: JASN Aug. 2023;1(8):1305–14. 10.1681/asn.0000000000000152. 10.1681/ASN.0000000000000152 PMC10400102 37131279

[39] Matthews AA, Danaei G, Islam N, Kurth T. Target trial emulation: applying principles of randomised trials to observational studies. BMJ Aug. 2022;30:378:e071108. 10.1136/bmj-2022-071108. 10.1136/bmj-2022-071108 36041749

[40] Wang SV, Schneeweiss S, Franklin JM, et al. Emulation of randomized clinical trials with nonrandomized database analyses: results of 32 clinical trials. JAMA: J Am Med Association Apr. 2023;25(16):1376–85. 10.1001/jama.2023.4221. 10.1001/jama.2023.4221 PMC10130954 37097356

[41] Antoine A, Pérol D, Robain M, Delaloge S, Lasset C, Drouet Y. Target trial emulation to assess real-world efficacy in the epidemiological strategy and medical economics metastatic breast cancer cohort. Journal Natl Cancer Institute Aug. 2023;8(8):971–80. 10.1093/jnci/djad092. 10.1093/jnci/djad092 PMC10407701 37220893

[42] Desai RJ, Franklin JM. Alternative approaches for confounding adjustment in observational studies using weighting based on the propensity score: a primer for practitioners. Bmj Oct. 2019;23:367:l5657. 10.1136/bmj.l5657. 10.1136/bmj.l5657 31645336

[43] Bang H, Robins JM. Doubly robust estimation in missing data and causal inference models. Biometrics. 2005;61(4):962–73. 10.1111/j.1541-0420.2005.00377.x. 16401269 10.1111/j.1541-0420.2005.00377.x

[44] Breskin A, Edmonds A, Cole SR, et al. G-computation for policy-relevant effects of interventions on time-to-event outcomes. Int J Epidemiol. 2021;23(6):2021–9. 10.1093/ije/dyaa156. 10.1093/ije/dyaa156 PMC7825964 33141177

[45] Lipsky AM, Greenland S. Causal directed acyclic graphs. JAMA: J Am Med Association Mar. 2022;15(11):1083–4. 10.1001/jama.2022.1816. 10.1001/jama.2022.1816 35226050

[46] Curtis LH, Sola-Morales O, Heidt J, et al. Regulatory and HTA considerations for development of real-world data derived external controls. Clin Pharmacol Ther. 2023;114(2):303–15. 10.1002/cpt.2913. 37078264 10.1002/cpt.2913

[47] Rosenbaum PR, Rubin DB. The central role of the propensity score in observational studies for causal effects. Biometrika. 1983;70(1):41–55. 10.2307/2335942.

[48] Austin PC, Schuster T. The performance of different propensity score methods for estimating absolute effects of treatments on survival outcomes: a simulation study. Stat Methods Med Res. 2016;25(5):2214–37. 10.1177/0962280213519716. 24463885 10.1177/0962280213519716 PMC5051602

[49] Schneeweiss S. Sensitivity analysis and external adjustment for unmeasured confounders in epidemiologic database studies of therapeutics. Pharmacoepidemiol Drug Saf. 2006;15(5):291–303. 10.1002/pds.1200. 16447304 10.1002/pds.1200

[50] VanderWeele TJ, Ding P. Sensitivity analysis in observational research: introducing the E-value. Ann Intern Med. 2017;15(4):268–74. 10.7326/m16-2607. 10.7326/M16-2607 28693043

[51] Sammon CJ, Leahy TP, Gsteiger S, Ramagopalan S. Real-world evidence and nonrandomized data in health technology assessment: using existing methods to address unmeasured confounding? J Comp Eff Res. 2020;9(14):969–72. 10.2217/cer-2020-0112. 32757772 10.2217/cer-2020-0112

[52] Leahy TP, Kent S, Sammon C, et al. Unmeasured confounding in nonrandomized studies: quantitative bias analysis in health technology assessment. J Comp Eff Res. 2022;11(12):851–9. 10.2217/cer-2022-0029. 35678151 10.2217/cer-2022-0029

[53] Lipsitch M, Tchetgen Tchetgen E, Cohen T. Negative controls: a tool for detecting confounding and bias in observational studies. Epidemiology. 2010;21(3):383–8. 10.1097/EDE.0b013e3181d61eeb. 20335814 10.1097/EDE.0b013e3181d61eeb PMC3053408

[54] Arnold BF, Ercumen A. Negative control outcomes: A tool to detect bias in randomized trials. JAMA: J Am Med Association Dec. 2016;27(24):2597–8. 10.1001/jama.2016.17700. 10.1001/jama.2016.17700 PMC5428075 28027378

[55] Rubin DB. Formalizing subjective notions about the effect of nonrespondents in sample surveys. J Am Stat Assoc. 1977;72(359):538–43. 10.2307/2286214.

[56] van Buuren S, Boshuizen HC, Knook DL. Multiple imputation of missing blood pressure covariates in survival analysis. Stat Med. Mar 1999;30(6):681–94. 10.1002/(sici)1097-0258(19990330)18:6%3C681::aid-sim71%3E3.0.co;2-r. 10.1002/(sici)1097-0258(19990330)18:6<681::aid-sim71>3.0.co;2-r 10204197

[57] Resseguier N, Giorgi R, Paoletti X. Sensitivity analysis when data are missing not-at-random. Epidemiology. 2011;22(2):282. 10.1097/EDE.0b013e318209dec7. 21293212 10.1097/EDE.0b013e318209dec7

[58] Leacy FP, Floyd S, Yates TA, White IR. Analyses of sensitivity to the Missing-at-Random assumption using multiple imputation with delta adjustment: application to a Tuberculosis/HIV prevalence survey with incomplete HIV-Status data. American J Epidemiology Feb. 2017;15(4):304–15. 10.1093/aje/kww107. 10.1093/aje/kww107 PMC5860630 28073767

[59] Wilkinson S, Gupta A, Scheuer N, et al. Assessment of alectinib vs ceritinib in ALK-positive non-small cell lung cancer in phase 2 trials and in real-world data. JAMA Netw Open. 2021;1(10):e2126306. 10.1001/jamanetworkopen.2021.26306. 10.1001/jamanetworkopen.2021.26306 PMC8498851 34618040

[60] Popat S, Liu SV, Scheuer N, et al. Addressing challenges with real-world synthetic control arms to demonstrate the comparative effectiveness of Pralsetinib in non-small cell lung cancer. Nat Commun. 2022;(1):3500. 10.1038/s41467-022-30908-1. 35715405 10.1038/s41467-022-30908-1 PMC9205915

[61] de Pouvourville G, Cunningham D, Fricke FU, et al. Across-country variations of real-world data and evidence for drugs: a 5-European-country study. Value Health. 2023. 10.1016/j.jval.2023.01.009. 36709042 10.1016/j.jval.2023.01.009

[62] Shift Health. When Decision Meets Precision: Evolving Health Technology Assessments for the Evaluation of Precision Oncology Therapeutics. Accessed 20 february 2024. https://www.shifthealth.com/insights/when-decision-meets-precision-evolving-health-technology-assessments-for-the-evaluation-of-precision-oncology-therapeutics/

[63] Evans WK, Takhar P, McDonald V, et al. Engaging patients in the Canadian Real-World evidence for value in cancer drugs (CanREValue) initiative: processes and lessons Learned. *Current oncology (Toronto, Ont)*. Aug. 2022;7(8):5616–26. 10.3390/curroncol29080443. 10.3390/curroncol29080443 PMC9406430 36005181

[64] Makin C, Sweet B, Rosenlund M et al. Solving the Real-World Evidence Challenge for US Payers: A Call to Action for Pharma. Value and outcomes Spotlight. January/February. 2024 2024;10(1).

[65] The DARWIN EU Coordination Centre. DARWIN EU. Accessed 7 March 2025. https://www.darwin-eu.org/index.php

[66] G_NIUS - Ministère. de la Santé et de la Prévention. Health Data Hub (HDH). Accessed 20 February 2024. https://gnius.esante.gouv.fr/en/players/player-profiles/health-data-hub-hdh

[67] Campbell UB, Honig N, Gatto NM. SURF: a screening tool (for sponsors) to evaluate whether using real-world data to support an effectiveness claim in an FDA application has regulatory feasibility. Clin Pharmacol Ther. 2023;114(5):981–93. 10.1002/cpt.3021. 37550832 10.1002/cpt.3021

[68] European Comission. Regulation on Health Technology Assessment. https://health.ec.europa.eu/health-technology-assessment/regulation-health-technology-assessment_en

